# Marula [*Sclerocarya birrea* (A. Rich.) Hochst.] products as a food and medicine

**DOI:** 10.3389/fphar.2025.1552355

**Published:** 2025-02-18

**Authors:** Beata Olas

**Affiliations:** Department of General Biochemistry, Faculty of Biology and Environmental Protection, University of Lodz, Lodz, Poland

**Keywords:** marula, marula fruit, marula juice, marula seed, antioxidant

## Abstract

The fruit of *Sclerocarya birrea* (A. Rich.) Hochst., commonly known as the marula, is widely appreciated for its nutritious pulp and edible nuts. The pulp has a higher vitamin C than those of other fruits, including pineapple, guava, and oranges. In addition, fresh marula fruits are often used to produce delicious sweets, wine and flavorings: it is perhaps best known as the flavor of Amarula liqueur. *In vitro* and *in vivo* studies indicate that the various parts of marula have pro-health properties, such as antioxidant, antibacterial, antifungal, antidiabetic activities. This paper reviews the current state of knowledge regarding the marula fruit and its products, with a special emphasis on their chemical composition, biological activity and pro-health potential.

## Introduction

While *Sclerocarya birrea* (A. Rich.) Hochst. has many common names, such as morula, jelly plum, cat thorn, cider tree, maroola plum, maroola nut, moroela, elephant tree, mafula, and nkanyi, it is best known as the marula. The species belongs to Anacardiaceae, alongside the sumac, cashew, mango and pistachio. *S. birrea* is divided into three subspecies differentiated by leaf shape and size: subsp. *birrea*, subsp. *afra* and subsp. *multifoliolata*. Subsp. *birrea* is found in northern Africa and subsp. *afra* in southern Africa, while subsp. *multifoliolata* is only found in Tanzania. The generic name *Sclerocarya* is derived from the Ancient Greek words *skleros* meaning *hard*, and *karyon* meaning *nut*, i.e., the hard pit of the fruit. The specific epithet *birrea* comes from the common name *birr*, for this type of tree in Senegal (Masarirambi and Nxumalo, 2012; Nyoko et al., 2015; Mashau et al., 2022; Abdelwahab et al., 2025).

The single-stemmed marula tree is found in 29 countries. Female trees bear up to 500 kg of fruit each year, while the male tree puts on a delicate floral display instead. Marula fruit are cylinder-like plum-sized drupes, and may range from 3 to 4 cm in thickness. They ripen between December and March, and have a light yellow skin with white flesh. Marula fruit is especially appreciated for its nutritious pulp and edible nuts. The pulp has a higher vitamin C (67–403 mg/100 g fresh weight) than the that of other fruits, including pineapples, guava, and oranges (Hiwilepo-van Hal, 2013). The ripe fruit is aromatic with a turpentine taste and the white succulent pulp sticks tightly to the nut (2–3 cm in diameter). The nut itself is divided into three or four hollows, each with one seed. The fruit has a single seed, which has a delicate nutty flavor (Mashau et al., 2022).

**FIGURE 1:**
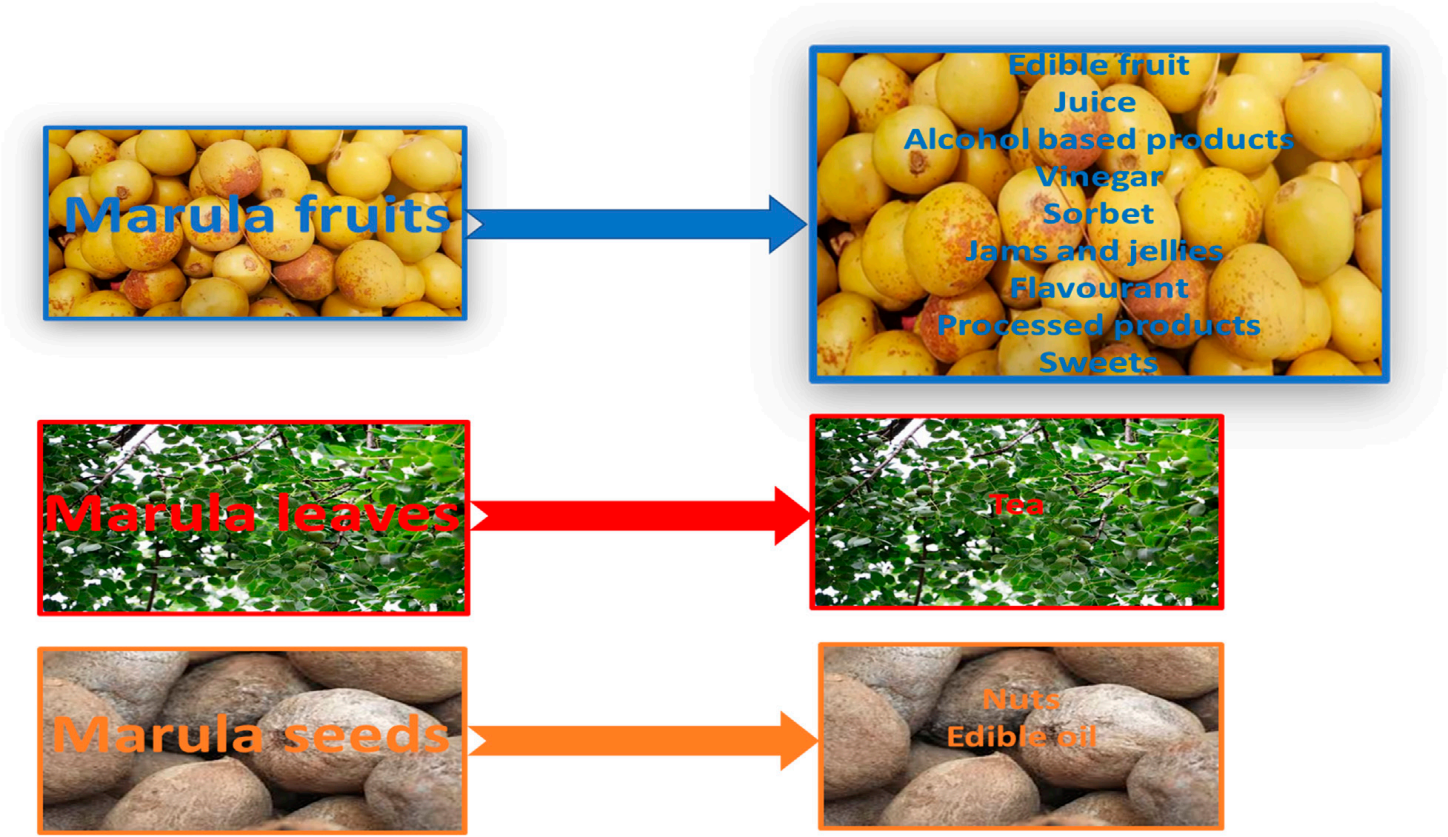
Bioactive ingredients of various parts of marula used as food.

The marula tree is also regarded as the “king of African trees”. It is a green, leafy plant standing between 9 and 18 m tall and demonstrates considerable drought resistance, with each tree still managing to produce large amounts of fruit during dry seasons. All of its parts, viz. the fruits, bark, leaves, stem, and nuts, are used extensively, with their precise use varying with location and tribe. The parts used for food are summarized in Figure 1. The fresh fruits can also be incorporated in various other products including wine, known locally as *mokhope*, *omagongo* or *ubuganu*, and flavorings, most famously Amarula liqueur. It is also used to produce delicious sweets similar to fruit rolls (Van Wyk, 2011; Pfukwa et al., 2020; Mashau et al., 2022).

**FIGURE 2:**
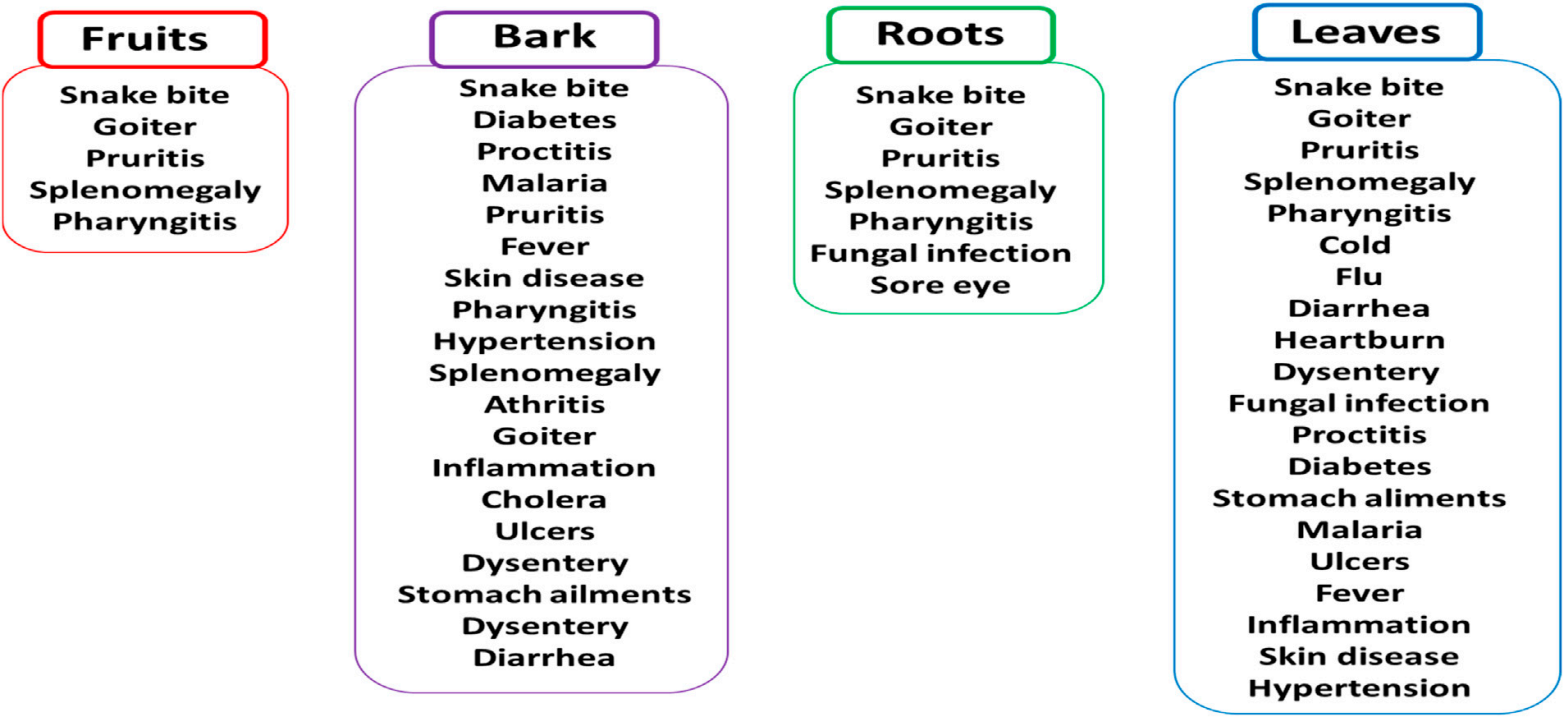
Marula parts (fruits, bark, roots, and leaves) used for treatment of pathology in folk medicine.

Marula fruits have demonstrated various antioxidant, antibacterial, antifungal and antidiabetic activities, as noted in *in vitro* and few *in vivo* studies (Pfukwa et al., 2020; Mashau et al., 2022; Fakudze et al., 2023; Abdelwahab et al., 2025); as such, they are often used in folk medicine (Figure 2). Although relatively few review papers have explored the medicinal potential of marula fruits (Mashau et al., 2022; Fakudze et al., 2023; Abdelwahab et al., 2025), their findings generally suggest the fruits have little medicinal value; however, these papers generally do not include the other parts of the marula plant and their products (Mashau et al., 2022; Lekhulenie et al., 2024). For example, a review by Mashau et al. (2022) only describes the biological activities of marula fruits.

The aim of the present mini-review is to provide an overview of the beneficial potential of marula fruits, and to highlight that other parts of the marula tree, and their products, also have beneficial potential.

## Research methods

ScienceDirect, Web of Science, SCOPUS, Web of Knowledge, PubMed, and Sci Finder were searched for papers examining potentially beneficial functional components of the various parts of the marula plant. The search terms comprised the terms “marula”; “*S. birrea*”, “marula tree”, “marula seed”, and “marula fruit” and their combinations. No time criteria were applied to the search, but recent papers were evaluated first. The last search was run on 15 December 2024. Papers were first selected based on the relevance to the title of the present manuscript, and the identified articles were screened by reading the abstract. Any relevant identified articles were summarized. About 88 articles were obtained from the searches, and only 43 were included in this review. The molecular mechanisms underpinning the biological action of various parts of marula were also analyzed and discussed as part of the search.

### Bioactive compounds of various parts of marula and marula products

Marula fruits contain considerable amounts of protein, dietary fiber, fatty acids, amino acids (threonine, tyrosine, methionine, valine, phenylalanine, isoleucine, leucine, lysis, and histidine), minerals (sodium, potassium, calcium, magnesium, copper, manganese, iron, and other), and vitamins (A, B_3_, C, E, and carotene) (Sibiya et al., 2020; Lekhulenie et al., 2024). The fruits also contain various other bioactive compounds, including phenolic compounds, phytosterols and triterpenoids, which determine their various biological properties (Mawoza et al., 2010; Hiwilepo-van Hal, 2013; Mashau et al., 2022; Lekhuleni et al., 2024).

Marula fruits may be eaten fresh or fermented into beer. The preparation of marula beer does not require additional substances such as sorghum, maize, yeast, and sugar (Molelekoa et al., 2018). The alcoholic concentration increases from 0.9% v/v, in the initial stage of fermentation, to 5.5% v/v in the final stage (Hiwilepo-van Hal, 2013). The fruit is also used to make a popular wine with high vitamin C and alcohol content, with the latter reaching strengths of 15%, depending on the tree and the length of fermentation (Hailwa, 1998; Hillman et al., 2008). The marula fruit is also, most famously, the base of the alcoholic Amarula cream liqueur, and milk chocolate with amarula syrup filling (Mashau et al., 2022; Lekhuleni et al., 2024). Marula fruits may be used to produce vinegar, which is often added to salad dressing and mayonnaise (Mashau et al., 2022).

Marula jams and jellies with an attractive natural waxy yellow color are also popular with consumers (Hiwilepo-van Hal, 2013; Mashau et al., 2022; Lekhuleni et al., 2024). Marula jam and ice cream manufactured according to industrial protocols were found to be rich in ascorbic acid 45 days post-production (Hillman et al., 2008). Various other marula products also exist, such as chutneys and pie fillings, fruit teas and supplements, i.e., powder capsules, as well as coffee substitute made from the burnt skin (Mawoza et al., 2010; Hiwilepo-van Hal, 2013; Mashau et al., 2022).

Marula juice prepared from fresh and fully-ripe pulp has high levels of vitamin C and various minerals, including manganese, potassium, zinc, calcium, and magnesium. Its sugar content is 22 mg/mL fructose, 4.9 mg/mL sucrose, and 4.9 g/mL glucose (Eromosesle et al., 1991; Hillman et al., 2008; Mashau et al., 2022). Hiwilepo-van Hal et al. (2013) report that the vitamin C in marula fruit pulp demonstrated considerably low thermal degradation. It was found to be 15 times more stable compared to guava and mango pulp.

Marula juice is also a good source of phenolic compounds including tannins, hydroxycinnamic acid derivatives and catechins (Borochov-Neori et al., 2008).

Marula seeds may be eaten dried, fresh or milled. In addition, they may be also incorporated to soups, boiled meat, and vegetables to enhance the flavor. Fresh marula seeds are also often mixed with porridge. De-kernelled marula seeds, including the shells, can be served as a tea or extract as a nutritional supplement (Pfukwa et al., 2020).

Importantly, marula seeds also contain various vitamins and minerals. They also contain oil which is a rich source of protein and mono-unsaturated fatty acids, especially oleic acid (Mariod et al., 2008; Mariod and Abdelwahab, 2012; Sybille et al., 2012; Mashau et al., 2022). However, Eromosele and Paschal (2003) indicate that marula oil has low concentration of vitamin E compared to other nut oils. A recent study comparing the effects of decoction and maceration on the phytochemical content and antioxidant properties of de-kernelled marula seeds by Rama et al. (2023) found decoction to achieve the highest concentrations of chemical compounds.

The varied uses of marula in food are presented in more detail in Figure 1. Table 1 also compared the nutritional content of edible marula fruit (pulp) and its products (nuts, jam and alcohol). It can be seen that pulp has higher fiber content (4.25–10.5 g/100 g) than the nut (2.47 g/100 g), lipids and protein levels are lower in the pulp than the nut. Again, it is important to note that marula pulp has a high concentration of vitamin C (0.062–0.179 g/100 g). In addition, marula pulp and nut are rich in various saturated fatty acids, such as tetradecanoic and hexadecanoic acid, and unsaturated fatty acids, such as oleic acid, linoleic acid, α-linolenic acid, and eicosanoid acid; the latter have pro-health properties, such as cardioprotective potential (Mariod and Abdelwahab, 2012; Sybille et al., 2012; Mashau et al., 2022).

**TABLE 1:** Concentrations of chemical compounds in marula fruit (pulp) and its products; and concentrations of phenolic compounds in various parts of marula (based on (Mashau et al., 2022); modified).

Chemical compound	Concentration of chemical compound (g/100 g)				References
	Marula pulp	Marula nut	Marula alcohol	Marula jam	
Proteins	12.5–30.1	26.5–28.4	—	—	Attiogbe and Abdul-Rozak (2016), Mashau et al. (2022), Chiesa et al. (2020)
Lipids	9.7–25.3	28.4–57.2	—	—	Attiogbe and Abdul-Rozak (2016), Mashau et al. (2022)
Carbohydrates	25.3 - 61.7	6.4–7.3	—	—	Hillman et al. (2008), Attiogbe and Abdul-Rozak (2016), Mashau et al. (2022)
Fiber	4.2–10.5	0 - 2.5	—	—	Eromosele et al. (1991), Lekhuleni et al. (2024)
β- caroten	0–0.1	-	—	—	Lekhuleni et al. (2024)
Vitamin C	0.062–0.179	-	—	—	Lekhuleni et al. (2024)
Calcium	0.006–0.052	0.106–0.156	—	—	Lekhuleni et al. (2024)
Magnesium	0.010–0.167	0.193–0.467	—	—	Lekhuleni et al. (2024)
Potassium	0.044–0.133	0–0.677	—	—	Lekhuleni et al. (2024)
Sodium	0.003–0.015	-	—	—	Lekhuleni et al. (2024)
Copper	0–0.001	0–0.002	—	—	Lekhuleni et al. (2024)
Iron	0–0.009	0.264–0.677	—	—	Lekhuleni et al. (2024)
Zinc	0–0.003	0.003–0.006	—	—	Lekhuleni et al. (2024)

Part of marula	Concentration of phenolic compounds	
	Total phenolic compounds (mg gallic acid equivalent (GAE)/100 g)	Flavonoids (mg quercetin equivalent (QE)/100 g)
Pulp	2.5–872.3	2.0–33.9
Leaves	13.9–304.5	0.47
Bark	23.0–593.0	No data
Roots	26.5–78.6	No data

The phenolic profile of marula varies with the part of the plant. For example, higher levels of flavonoids are noted in marula pulp than the leaves, bark, and roots (Table 1). The polyphenolic content of marula fruit is described in more detail by Mashau et al. (2022) and Lekhuleni et al. (2024).

### Chemical anti-oxidant assays for marula products

Hillman et al. (2008) noted that fresh marula fruit juice has antioxidant capacity ranging from 141–440 mg/100 mL ascorbic acid equivalent (*in vitro* model). The total antioxidant and polyphenol content of the juice varied among clones and with time post-abscission, as did the activity of superoxide dismutase. In addition, ascorbic acid content was found to be high in all the clones with significant differences among clones and time post-abscission. Clone no 12 at 1 week post-abscission had the higher ascorbic acid content, 21.177 mg/g dry weight and clone no four at the abscission day had the lower ascorbic acid content, 7.142 mg ascorbic acid/g dry weight. It also has around four times more antioxidant potential than the juice of either orange or pomegranate. In addition, marula ice cream and jam manufactured according to industrial protocols were rich in ascorbic acid 45 days post-production.

Mariod et al. (2008) studied radical scavenging capacities and antioxidant properties of various methanolic extracts from *S. birrea* leaves, roots, barks, and kernel oil cake *in vitro*. The total phenolic compounds were found as 304.5 mg/g of dry product (for the extract from leaves), 367.5 mg/g of dry product (for the extract from roots), 593.0 mg/g of dry product (for the extract from barks), and 258.0 mg/g of dry product (for the extract from kernel oil cake). All tested extracts were markedly effective in inhibiting the oxidation of linoleic acid and subsequent bleaching of β-carotene in comparison with the control. However, based on oxidation of β-carotene ⁄ linoleic acid, the extract from kernel oil cake was the most effective followed by extracts from roots, leaves, and barks. The antioxidant properties determined by the DPPH (1,1-diphenyl-b-picrylhydrazyl) method also revealed that the extract from kernel oil cake had the highest antioxidant activity on DPPH free radicals followed by extracts from barks, roots, and leaves.

Other research found young stem extract of marula to contain the highest levels of total phenolic compounds (14.15 ± 0.03 mg gallic acid equivalent (GAE)/g), flavonoids (1.21 ± 0.01 mg catechin equivalent (CE)/g) and gallotannins (0.24 ± 0.00 mg GAE/g) compared to other parts. The EC_50_ value of the stem extract was found to be 5.02 μg/mL (6.86 μg/mL for ascorbic acid) in the DPPH free radical test (Moyo et al., 2010). Also, the antioxidant properties of extracts from marula leaves, bark and roots have been associated with their phenolic compound content (Mariod et al., 2008; Moyo et al., 2010; Misihairabgwi and Cheikhyoussef, 2017).

In addition, Ndhlala et al. (2006a) and Ndhlala et al. (2006b) report that marula juice has antioxidant potential associated with its phenolic compounds (total phenolics: 2262 µg GAE/g, flavonoids: 202 µg catechin/g, and 6.0% condensed tannins). Other studies attribute the antioxidant properties of marula juice to the degree of polymerization (DP) of phenolic compounds, when DP is less than 10 (Zhou et al., 2014).

A recent study of the effect of freezing on the chemical content (total phenolic compounds: 196.42 mg catechin equivalent/mL; 0 weeks) and antioxidant properties of marula fruit juice by Nthabising et al. (2023) found an approximate 37% decrease in total phenolic compounds, and 36% decrease in vitamin C compared to fresh, unfrozen controls as a result of the freezing process; this data indicates that freezing is not a good method of preservation. Freezing also reduced the antioxidant properties of the fruit juice, measured by various methods.

### Biological activity of marula

Marula products and supplements have commercial, cultural and ethnomedicinal value in Africa and in other parts of the world. However, studies indicate that the different parts of the plant have different biological properties.

#### Antioxidant potential (*in vitro* and *in vivo* models)

Borochov-Neori et al. (2008) indicate that marula juice (1 and 2 μg/mL) showed radical scavenging capacity by causing a 32% and 62% reduction, in optical density of DPPH solution. In a model of oxidative stress caused by copper ions, marula juice also decreased the production of lipid peroxide (IC_50_–0.055 μg/mL) and thiobarbituric acid reactive substances (TBARS) (IC_50_–0.050 μg/mL) caused by LDL oxidation. Moreover, supplementation of this juice (100 and 200 mL/kg b.w./day; for 3 weeks) significantly reduced the level of triglyceride (7%), LDL (17%), and total cholesterol (8%), and increased the level of high-density lipoprotein (HDL) by 10% in healthy individuals. The juice contained a significant amount of phenolic compounds (56 mg of pyrogallol equivalence) and its antioxidant capacity was found to be high (382 mg of ascorbic acid equivalent). Most importantly, its antioxidant activity was not destroyed by pasteurization; however, after 4 weeks of low temperature storage (at – 18 °C), the antioxidant potential was reduced by 14%. In addition, tested marula juice was found to contain high vitamin C and potassium levels and low sugar concentration (267 mg/dL, 328 mg/dL, and 7.3 g/dL, respectively).

#### Antidiabetic potential (*in vivo* models)

Various studies have reported that marula bark extract has antidiabetic activity in animal models. For example, Gondwe et al. (2008) observed that 5-week supplementation modulates blood glucose and glomerular filtration rate in diabetic rats. The extract was found to have the same effects as methormin treatment. The action of used extract (120 mg/kg p.o., daily) was monitored for 5 weeks in animals. This extract exhibited dose-dependent reduction in blood glucose concentration. In addition, the hypoglycemic effect of this extract treatment was associated with increased hepatic glycogen synthesis. Moreover, the extract treatment reduced blood pressure in animals.

Dimo et al. (2007) also noted that administration of marula bark extract reduces the level of blood glucose in diabetic rats. Experimental animals using by Dimo et al. (2007), were treated by oral administration of plant extract (150 and 300 mg/kg body weight) and metformin (500 mg/kg; control - reference drug) for 21 days. They observed that marula stem bark extract exhibited a significant reduction in blood glucose and increased plasma insulin levels in diabetic rats. Moreover, used extract also prevented body weight loss in diabetic rats. The effective dose of this extract (300 mg/kg) tended to reduce plasma cholesterol, triglyceride and urea levels toward the normal levels.

#### Antibacterial and antifungal potential (*in vitro* models)

Eloff (2001) report that marula bark and leaf extracts have antibacterial properties against *Staphylococcus aureus*, *Pseudomonas aeruginosa*, *Escherichia* faecais, and *Escherichia* cola. The minimum inhibitory concentration (MIC) values ranged from 0.15 to 3 mg/mL.

Interestingly, various parts of the marula also have antifungal activity. For example, Masoko et al. (2008) found acetone, ethanol, and methanol marula bar extracts to have antifungal properties against *Candida* parapsilosis, Cryptococcus albidus, and Rhodotorula mucilaginosa. Of the tested extracts, the greatest activity was found for methanol. Hamza et al. (2006) also found marula root methanolic extract to have antifungal activity against Cryptococcus neoformans, *Candida* albicans, *Candida* kruseii, *Candida* glabrata, *Candida tropicalis*, and *Candida* parapsilosis with a MIC value of 0.5 mg/mL. However, authors (Eloff, 2001; Hamza et al., 2006; Masoko et al., 2008) did not describe the phytochemical profile of used extracts.

#### Other biological potential (*in vitro* and *in vivo* models)

The anti-inflammatory effects of aqueous and methanolic stem-bark extracts of S. birrea (500 mg/kg p.o.) were also examined on rat paw oedema induced by subplantar injections of fresh egg albumin (0.5 mL/kg). The typical anti-inflammatory agent - acetylsalicylic acid (100 mg/kg p.o.) was used for comparison. Both, the aqueous and methanolic extracts progressively and time-dependently reduced rat paw oedema induced by subplantar injections of fresh egg albumin. But, the methanolic extract produced relatively greater and more pronounced anti-inflammatory effect than its aqueous extract counterpart in the experimental animal model (Ojewole, 2003).

A combination of marula stem bark extract and those of other medicinal trees was tested against Plasmodium falciparum and Plasmodium berghei. The extracts demonstrated both anti-plasmodial activity *in vitro* and anti-malarial potential *in vivo*. However, phytochemical profile of the tested extract was not described (Gathirwa et al., 2008).

A recent study found marula oil nanoemulsion to have neuroprotective potential in a mouse model of experimental Parkinson’s disease induced by rotenone (Alshaman et al., 2023). Treatment with marula oil or marula nanoemulsion improved poor motor performance; it also downregulated the elevated expression of protein inflammatory parameters, such as tumor necrosis factor-α (TNF-α) and interleukin-1β (IL-1β) in the mice, as well as biomarkers of oxidative stress (e.g., TBARS).

Recently, Tientcheu et al. (2023a) and Tientcheu et al. (2023b) also investigated the neuroprotective and antidiabetic properties of a mixture of Piper longum, Nauclea latifolia, and S. birrea (75, 150, and 300 mg/kg) in a rat model of type 2 diabetes and memory impairment. The tested mixture (150 and 300 mg/kg) showed neuroprotective and memory-strengthening potential by protecting the hippocampus neurons and normalized blood glucose levels. Other studies by these authors indicate that this mixture may protect the striatal neurons and movement-associated functionalities in a rat model of locomotion dysfunction caused by diabetes. However, authors only describe that tested mixture contains active metabolites, mainly flavonoids, and fatty acids.

**FIGURE 3:**
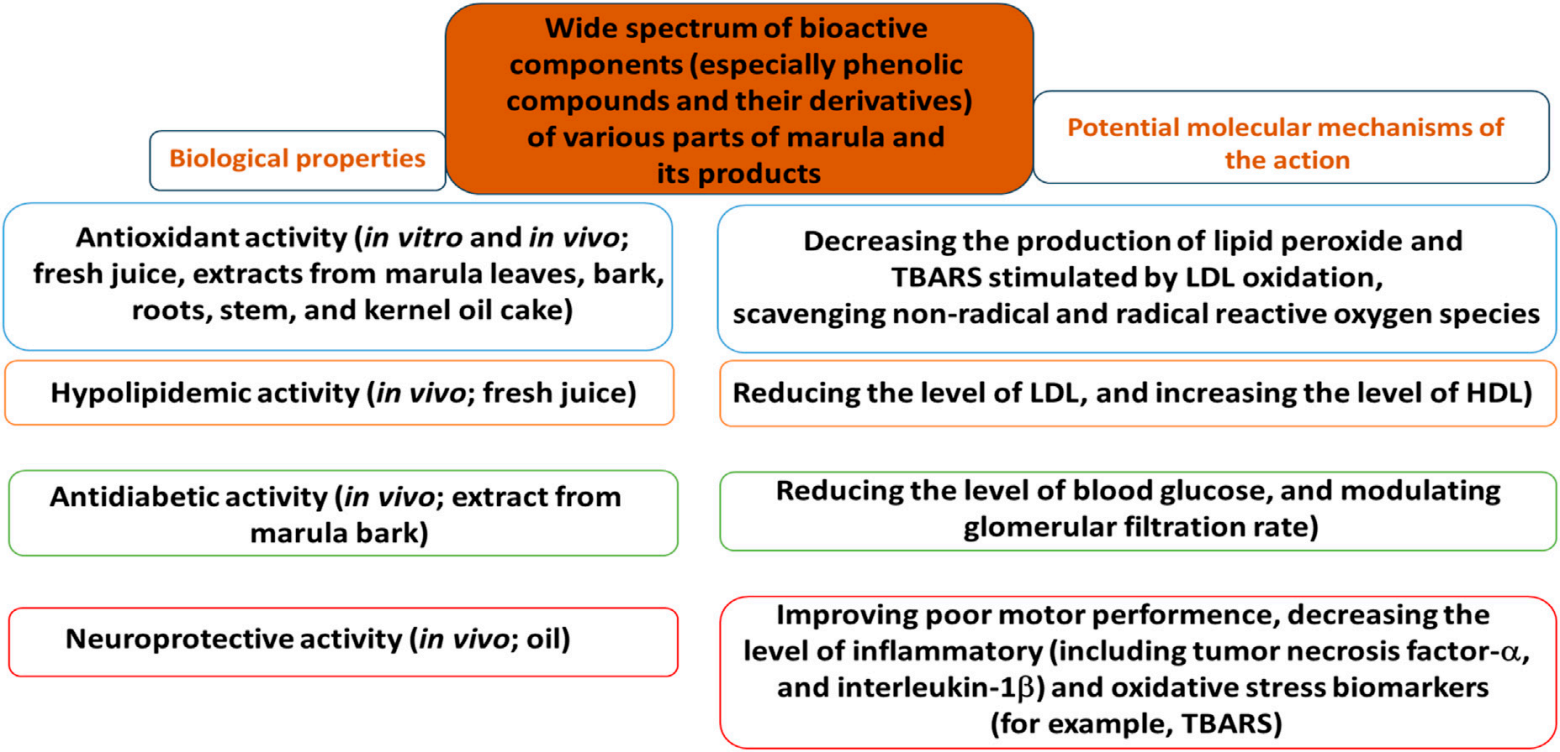
Main biological properties of various parts of marula and its products; their potential molecular mechanims

Wide spectrum of active components, especially phenolic compounds of various parts of marula and its products have been found to demonstrate different biological properties and these are summarized in Figure 3 together with their potential molecular mechanisms. For example, marula juice and extracts from various parts of marula have been noted to effectively inhibit generation of non-radical and radical reactive oxygen species and lipid peroxidation. In addition, marula oil demonstrates neuroprotective activity, which may be associated with its anti-inflammatory effect by regulating signaling pathways such as TNF-α and IL-1β.

### The safety of marula extracts

Some *in vitro* and animal studies have examined the safety of marula extracts, but with varying results. For example, Muhammad et al. (2014) report in in vivo model that marula fruit peel extract (oral administration: 3000 and 4,000 mg/kg body weight) has a toxic effect on kidneys and liver in rats, manifested in significantly higher uric acid, urea, serum total protein, creatinine, transaminase, bilirubin, and albumin content.

An *in vitro* study found that exposure to high concentrations (600–1000 μg/mL) of bark-stem extract decreases the viability of kidney cell lines (distal and proximal tubules) (Gondwe et al., 2008), but the same extract (120 mg/kg) has no effect on renal fluid in nondiabetic and diabetic rats.

The cytotoxicity of marula was tested against VERO cells (*in vitro*), evaluated as the concentration required to cause visible alterations in 50% of intact cells (CC_50_). The analysis found the methanolic extract to have a CC_50_ of 361.2 μg/mL and the aqueous extract 3375.2 μg/mL. However, the authors did not write which part of marula was used (Gathirwa et al., 2008).

It has also been found *in vivo* that aqueous, methanolic, and hexane extracts from marula bark and stem (10–1000 mg/mL) did not demonstrate toxic potential against brine shrimp (McGaw et al., 2007). However, Ojewole (2023) report that methanolic and aqueous marula bark-stem extracts (500 mg/kg) are safe in rats. Unfortunately as there is not yet any unequivocal clinical evidence for the safety of marula products and supplements, further research is needed to confirm this. More details about the toxicity of marula extracts are described by Fakudze et al. (2023). However, this review paper only reports research data *in vitro* models.

## Conclusion

Marula is consumed in a variety of forms, including raw fruits, alcohol-based products, jams and jellies. Marula seeds are surrounded by a delicate white kernel high in arginine and glutamic acid, as well as myristic, oleic, and palmitic acids. Marula preparations (especially extracts) have high levels of total phenolic compounds, with various biological properties (Table 2). However, many of these studies are limited to *in vitro* and animal models, and demonstrate considerable heterogeneity, making it difficult to compare results. In addition, authors often do not describe the phytochemical profile of used preparations, including extracts. Often biological activities of marula preparations reported very naively. Moreover, such papers about biological properties of marula products are sometimes common in journals with no or poor peer-review. In addition, full taxonomic validity of the plant material under investigation was not always ascertained.

**TABLE 2:** Biological properties of various marula products.

Marula product	Biological activity	References
Fruit juice (100 and 200 mL/kg b.w., for 3 weeks; amount of phenolic compounds – 56 mg of pyrogallol equivalent)	Hypolipidemic activity (*in vivo –* healthy humans)	Borochov-Neori et al. (2008)
Extract from stem bark (500 mg/kg p.o.; non data – chemical content)	Anti-inflammatory activity (*in vivo* - rat paw oedema induced by subplantar injections of fresh egg albumin (0.5 mL/kg))	Ojewole (2003)
Extract from roots (0.5 mg/mL; non data – chemical content)	Antifungal activity (*in vitro*)	Hamza et al. (2006)
Extract from bark (120 mg kg(-1) p.o., daily), for 5 weeks, non data – chemical content)	Antidiabetic activity (*in vivo* – diabetic rats)	Gondwe et al. (2008)
Extract from bark (150 and 300 mg/kg body weight, daily, for 21 days); non data – chemical content)	Antidiabetic activity (*in vivo* – diabetic rats)	Dimo et al. (2007)
Extract from bark (0.15–3 mg/mL; non data – chemical content)	Antibacterial activity (*in vitro*)	Eloff (2001)

Although the food products derived from marula fruits and seeds may be valuable sources of bioactive compounds, further *in vivo* studies are needed to clarify their exact mechanism (Hiwilepo-van Hal et al., 2012; Hiwilepo-van Hal et al., 2014; Sibiya et al., 2020; Mashau et al., 2022; Lekhuleni et al., 2024). Such studies should also examine the health potential of fresh marula fruits, and their food products and supplements, as well as their long-term effects. Moreover, only few papers (Ojewole, 2003; Gathirwa et al., 2008; Gondwe et al., 2008; Muhammad et al., 2014; McGaw et al., 2007) describe safety of extracts from various parts of marula in in vitro and animal models. There are no information about the safety of food products derived from marula fruits and seeds in animal and human models. Such studies should examine the safety of these food products, as well as their long-term safety.

The biological activity of marula fruits and their products is doubtlessly influenced by their chemical composition, with vitamin C, phenolic compounds, and unsaturated fatty acids playing key roles. However, again, their mechanisms of action are unclear and require further study.

## Abbreviations

CE, catechin equivalent; DP, degree of polymerization; DPPH, 2,2-diphenyl-1-picrlhydrazyl; GAE, gallic acid equivalent; HDL, high-density lipoprotein; Il, interleukin; LDL, low-density lipoprotein; MIC, minimum inhibitory concentration; TBARS, thiobarbituric acid reactive substances; TNF-α, tumor necrosis factor-α.
